# Detecting Introgression Between Members of the *Fusarium fujikuroi* and *F. oxysporum* Species Complexes by Comparative Mitogenomics

**DOI:** 10.3389/fmicb.2020.01092

**Published:** 2020-06-03

**Authors:** Balázs Brankovics, Anne D. van Diepeningen, G. Sybren de Hoog, Theo A. J. van der Lee, Cees Waalwijk

**Affiliations:** ^1^B.U. Biointeractions and Plant Health, Wageningen Plant Research, Wageningen University & Research, Wageningen, Netherlands; ^2^Westerdijk Fungal Biodiversity Institute, KNAW, Utrecht, Netherlands; ^3^Center of Expertise in Mycology, Radboud University Medical Center, Nijmegen, Netherlands

**Keywords:** mitogenomics, introgression, *Fusarium oxysporum* species complex, *F. fujikuroi* species complex, phylogenetics, horizontal gene transfer

## Abstract

The *Fusarium fujikuroi* species complex (FFSC) and *F. oxysporum* species complex (FOSC) are two related groups of plant pathogens causing a wide diversity of diseases in agricultural crops world wide. The aims of this study are (1) to clarify the phylogeny of the FFSC, (2) to identify potential deviation from tree-like evolution, (3) to explore the value of using mitogenomes for these kinds of analyses, and (4) to better understand mitogenome evolution. In total, we have sequenced 24 species from the FFSC and a representative set of recently analyzed FOSC strains was chosen, while *F. redolens* was used as outgroup for the two species complexes. A species tree was constructed based on the concatenated alignment of seven nuclear genes and the mitogenome, which was contrasted to individual gene trees to identify potential conflicts. These comparisons indicated conflicts especially within the previously described African clade of the FFSC. Furthermore, the analysis of the mitogenomes revealed the presence of a variant of the large variable (LV) region in FFSC which was previously only reported for FOSC. The distribution of this variant and the results of sequence comparisons indicate horizontal genetic transfer between members of the two species complexes, most probably through introgression. In addition, a duplication of *atp9* was found inside an intron of *cob*, which suggests that even highly conserved mitochondrial genes can have paralogs. Paralogization in turn may lead to inaccurate single gene phylogenies. In conclusion, mitochondrial genomes provide a robust basis for phylogeny. Comparative phylogenetic analysis indicated that gene flow among and between members of FFSC and FOSC has played an important role in the evolutionary history of these two groups. Since mitogenomes show greater levels of conservation and synteny than nuclear regions, they are more likely to be compatible for recombination than nuclear regions. Therefore, mitogenomes can be used as indicators to detect interspecies gene flow.

## 1. Introduction

Members of the *Fusarium fujikuroi* species complex (FFSC) are plant pathogens causing a wide diversity of diseases in agricultural crops worldwide. The species are difficult to distinguish by morphological characters and often share a high (90%) sequence similarity, but can differ in host plant specificity, lifestyle and secondary metabolite production (Niehaus et al., 2016). Based on a biogeographic hypothesis proposed from the phylogenetic evidence, three major clades have been described within the FFSC: named as the African, American, and Asian clades (O'Donnell et al., 1998). Although multilocus sequence analysis supports the recognition of the three biogeographic clades, several genetic markers appear to contradict this grouping, such as the ITS2 region (O'Donnell et al., 1998, 2000) and the fumonisin biosynthetic gene cluster (Proctor et al., 2013). Interspecies recombination was suggested within the FFSC based on comparative mitochondrial genome analysis that found conflicting mitochondrial gene trees (Fourie et al., 2013, 2018).

A gene tree may deviate from the species tree, because of recombination, incomplete lineage sorting (ILS) (Zhou et al., 2017) and horizontal gene transfer (HGT) (Arvestad et al., 2003). ILS is the result of allelic polymorphism that is maintained after speciation, although the polymorphism may be lost through fixation in the daughter species. ILS can be identified by comparing the putative species tree and the gene trees based on the different allelic groups (Ward et al., 2002; Brankovics et al., 2017). Concordance between the species tree and the allelic trees indicates ILS; however, this is only possible when the polymorphism is still present in all or most daughter species included. HGT transfer can be detected by comparing the pairwise genetic distances of the putative transferred region and the genomic genetic distance (average distance of multiple regions) (Novichkov et al., 2004). When the gene based genetic distance is significantly lower than the genome based one, it indicates a possible HGT event. A common mechanism for HGT is introgression (or “introgressive hybridization”). Introgression is the incorporation of genes or alleles through hybridization and backcrossing between two distinctive species (Anderson and Hubricht, 1938; Anderson, 1949).

We have recently identified incomplete lineage sorting in the case of the large variable region of the mitochondrial genome of the *F. oxysporum* species complex (FOSC) (Brankovics et al., 2017). The mitogenomes of *Fusarium* spp. contain a set of fourteen “standard” mitochondrial polypeptide-encoding genes, two rRNA-encoding genes, *rnl* (mtLSU) and *rns* (mtSSU), and more than twenty tRNA-encoding genes (Al-Reedy et al., 2012). The genes, their orientation and order are highly conserved within the FOSC, except for a region located between *rnl* and *nad2*. This exceptionally variable region is referred to as large variable (LV) region, which in most cases harbors a set of tRNA-encoding genes and a large open reading frame (ORF) with unknown function (LV-uORF) (Al-Reedy et al., 2012; Fourie et al., 2013; Brankovics et al., 2017). In the case of *F. oxysporum*, this region has at least three forms or variants that show low levels of similarity to each other. Two of the variants are present in two of the major clades, the trees based on nuclear and mitochondrial markers are congruent with trees based on each of the variants, which indicates ILS (Brankovics et al., 2017).

Previous comparative mitogenome analyses of *F. fujikuroi* species complex (FFSC) by Fourie et al. (2013, 2018) have indicated that mitochondrial recombination is happening between species within this complex. Unfortunately, the first study only included one representative for each of the three major lineages, while the second one did not include the LV region, which limited the resolution of the analyses and the strength of the conclusions. Furthermore, these studies focused only on the mitogenomes and did not examine the evolution of nuclear markers. The aims of this study are (1) to clarify the phylogeny of the *F. fujikuroi* species complex (FFSC), (2) to identify potential deviation from tree-like evolution, (3) to explore the value of using mitogenomes for these kinds of analyses, and (4) to better understand mitogenome evolution. In total, 36 strains were analyzed: 24 strains from the FFSC and 9 representative strains were chosen from the set of recently analyzed FOSC strains (Brankovics et al., 2017), two strains that are closely related the FOSC, and *F. redolens* that was used as the outgroup. A species tree was constructed based on the concatenated alignment of seven nuclear genes and the mitogenome to serve as a reference. Individual gene trees were then compared to the species tree identifying conflicts that may indicate hybridization events, incomplete lineage sorting or horizontal gene transfer. The comparative mitogenomic analyses identify two insertion events: the duplication of a conserved gene, *atp9*, into another gene's intron, and the insertion of variant 2 of the large variable (LV) region into a variant 1 of the LV region. In addition, the phylogeny of the LV variant 2 sequences is indicative of horizontal transfer within the FFSC and between the FFSC and the FOSC.

## 2. Materials and Methods

### 2.1. Strains

In total 24 species belonging to the *F. fujikuroi* species complex (FFSC) were analyzed in this study, with 14 belonging to the African clade, 5 to the American clade, and 5 to the Asian clade of the FFSC (O'Donnell et al., 1998). In addition, nine strains were selected to represent the breadth and diversity of the *F. oxysporum* species complex (FOSC), and two sister species of the FOSC, *F. foetens* and *F. commune*, were analyzed. *F. redolens* was included as an outgroup to both the FFSC and the FOSC (Table 1).

**Table 1:** LV variant and intron presence in the analyzed strains.

**Sp. complex**	**Clade**			**Introns**																							
		**Strain**	**LV variant**	**atp6-i365**	**cob-i201**	**cob-i393**	**cob-i4**	**cob-i823**	**cox1-i218**	**cox1-i287**	**cox1-i392**	**cox1-i621**	**cox1-i715**	**cox1-i737**	**cox1-i873**	**cox1-i6**	**cox1-i1063**	**cox1-i1131**	**cox1-i1268**	**cox2-i228**	**cox3-i219**	**nad1-i636**	**nad2-i762**	**nad2-i1632**	**nad5-i717**	**nad5-i924**	**rnl-intron**	**GenBank accession numbers**
FFSC
	African
		***F. pseudoanthophilum*** **CBS414.97**	1		+	+	+		+	+	+	+	+		+		+		+		+			+		+	+	MT010924
		***F. brevicatenulatum*** **CBS404.97**	1		+	+	+		+	+	+	+	+		+		+		+	+	+			+		+	+	MT010923
		***F. napiforme*** **CBS748.97**	1		+	+	+			+	+	+	+		+		+		+					+		+	+	MT010918
		***F. ramigenum*** **CBS418.97**	2		+						+	+	+		+		+				+			+		+	+	MT010919
		***F. pseudonygamai*** **CBS417.97**	1		+	+	+	+			+	+	+		+		+		+					+			+	MT010925
		***F. ficicrescens*** **CBS125178**	2			+	+		+															+			+	MT010922
		***F. musae*** **CBS624.87**	1			+	+						+				+		+			+		+			+	MT010916
		***F. verticillioides*** **CBS576.78**	1										+				+					+		+			+	MT010915
		***F. andiyazi*** **CBS119857**	1									+												+			+	MT010917
		***F. nygamai*** **CBS749.97**	1												+				+			+		+			+	MT010926
		***F. lactis*** **CBS411.97**	2										+											+			+	MT010921
		***F. pseudocircinatum*** **CBS449.97**	1										+											+			+	MT010920
		***F. denticulatum*** **CBS407.97**	2			+							+	+	+		+	+						+			+	MT010934
	American
		***F. guttiforme*** **CBS409.97**	1			+	+		+			+	+		+	+	+	+	+			+	+	+			+	MT010931
		***F. ananatum*** **CBS118516**	2			+	+		+			+	+		+	+	+		+			+	+	+			+	MT010930
		***F. begoniae*** **CBS452.97**	1^b^			+	+		+			+	+		+	+	+		+			+	+	+			+	MT010929
		***F. anthophilum*** **CBS119858**	1									+	+		+	+	+					+	+	+			+	MT010928
		***F. bactridioides*** **CBS100057**	1			+	+		+		+	+	+		+	+	+	+	+	+	+	+	+	+			+	MT010927
	Asian
		***F. annulatum*** **CBS258.54**	2																								+	MT010912
		*F. proliferatum* ITEM2400	1																								+	LT841261
		***F. globosum*** **CBS428.97**	1																								+	MT010913
		***F. concentricum*** **CBS450.97**	1																								+	MT010911
		***F. sacchari*** **CBS147.25**	1			+	+		+		+		+	+										+			+	MT010910
	-^a^	***F. acutatum*** **CBS402.97**	1												+				+					+	+		+	MT010914
FOSC
		*F. oxysporum* Fol4287	2			+																			+		+	LT6324
	3	*F. oxysporum* F11	1			+																			+		+	LT841205
		*F. oxysporum* FOSC3a	2			+	+																		+		+	LT6345
	4	***F. oxysporum*** **CBS130302**	1			+																			+		+	MT010933
		*F. oxysporum* Fom009	3																						+		+	LT6337
	2	*F. oxysporum* Fon013	2																						+		+	LT6337
		*F. oxysporum* N2	1																						+		+	LT6350
	1	*F. oxysporum* II5	1	+		+	+																		+		+	LT6347
		*F. oxysporum* Foc011	1			+																			+		+	LT6308
		***F. foetens*** **CBS110286**	1																						+		+	MT010932
		*F. commune* JCM11502	1																			+			+		+	NC_036106
		***F. redolens*** **CBS743.97**	1											+			+			+							+	MT010909

*The strains are ordered according to the species tree (Figure 6A). F. acutatum belongs to the African clade sensu (O'Donnell et al., 1998), but it groups separately in the current analysis (shown as -^a^). The LV region of F. begoniae is variant 1 with a putative variant 2 insert (shown as 1^b^). Strains sequenced for this study are shown in bold*.

### 2.2. Sequencing

For each strain a random sheared shotgun library was prepared using NEXTflex ChIP-seq Library prep kit with few adaptations for low input gDNA according to manufacturer's protocol (Bioscientific). The library was loaded on an Illumina paired-end flowcell for cluster generation using a cBot. Sequencing was done on an Illumina HiSeq2000 instrument using 125, 7, 125 flow cycles for forward, index, and reverse reads respectively. De-multiplexing of resulting data was carried out using Casava 1.8 software. The 26 strains sequenced for this study are highlighted in Table 1. Sequencing data is available under the following accession number: PRJEB38038.

### 2.3. Assembly

The following (complete: from start to stop codon) nuclear protein coding genes were assembled from NGS data for all strains: γ-actin (*act*), β-tubulin II (*tub2*), calmodulin (*cal*), the largest and second largest subunit of DNA-dependent RNA polymerase II (*rpb1* and *rpb2*, respectively), translation elongation factor 1α (*tef1a*) and translation elongation factor 3 (*tef3*). These nuclear genes were selected for this study either because they are commonly used or have been suggested as good candidates for phylogeny and DNA barcoding for fungi (Stielow et al., 2015). Besides the nuclear protein coding genes, the complete mitochondrial genomes were also assembled for all strains.

The regions listed above were assembled from NGS reads using GRAbB (Genomic Region Assembly by Baiting; Brankovics et al., 2016) by specifying the appropriate reference sequence and employing SPAdes 3.6 (Bankevich et al., 2012; Nurk et al., 2013) as assembler.

### 2.4. Sequence Annotation

The initial mitogenome annotations were done using MFannot (http://megasun.bch.umontreal.ca/cgi-bin/mfannot/mfannotInterface.pl) and were manually improved: annotation of tRNA genes was improved using tRNAscan-SE (Pavesi et al., 1994), annotation of protein-coding genes and the *rnl* gene was corrected by aligning intronless homologs to the genome. Intron encoded proteins were identified using NCBI's ORF Finder (https://www.ncbi.nlm.nih.gov/orffinder/) and annotated using InterProScan (Mitchell et al., 2015).

### 2.5. Comparative Sequence Analysis

The nucleotide sequences were aligned using MUSCLE (Edgar, 2004a,b). Nuclear gene sequences were aligned per gene, while the mitochondrial sequences were aligned per region (exons, intergenic regions), and subsequently concatenated to yield the final alignment.

Genetic distances were calculated using the *ape* package (Paradis et al., 2004) of *R*: FASTA format sequence alignments were imported into *R*, pairwise distance were calculated using *dist.dna* function with standard settings. The *ggplot* function from the *ggplot2* package (Wickham, 2009) was used for plotting the distance data as a scatter plot. The *lm* function was used for linear regression, 0 intercept was used for the models.

In the mitogenome of *F. sacchari* a duplication was found involving *atp9*, therefore sequence repeats were identified using exonerate (Slater and Birney, 2005) by using the mitogenome of *F. sacchari* both as query and target. Hits were filtered, and repeats longer than 100 bp and scoring at least 80% of the possible maximal score were kept (settings were chosen for clarity of visualization). These hits were visualized using Circos (Krzywinski et al., 2009), which then was manually combined with the genetic map drawn using CLC Sequence Viewer.

### 2.6. Phylogenetic Analysis

Phylogenetic analyses were conducted using the ML method which was run using IQ-Tree (Nguyen et al., 2014) with model selection (Kalyaanamoorthy et al., 2017) and 1,000 ultrafast bootstraps (Hoang et al., 2017) and with the *F. redolens* as the outgroup. The phylogenetic trees were visualized using an in-house script *print-single-nwk.py* that is available on GitHub (https://github.com/b-brankovics/simple-nwk-tools). The comparison table between phylogenetic trees was generated using another in-house script *phylo-report.pl* that is available on GitHub (https://github.com/b-brankovics/phylo-report).

Clade monophyly was tested using the Shimodaira-Hasegawa (SH) test and the approximately unbiased (AU) test. In total, 34 constrain trees were specified: 33 trees with one branch from the reference tree specifying the constraint, also we added a constraint tree with the African clade. Each constrain tree was constructed for each alignment using IQ-tree. Then all the constrain trees per alignment were tested with the SH and AU tests built into IQ-tree. Finally, all results were collected and organized into Table S1.

## 3. Results

### 3.1. Phylogeny

**Figure 1:**
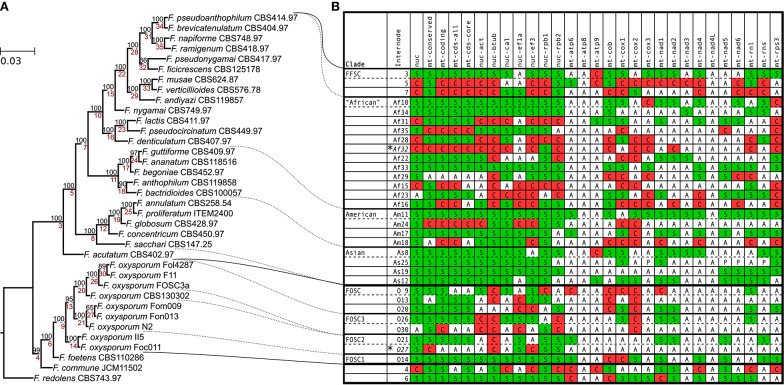
Species tree and internode compatibility with individual trees. **(A)** Species tree estimated based on the concatenated sequence of the conserved region of the mitocgenome and seven nuclear protein coding genes; bootstrap values are shown above the branches, and internode labels are indicated below the branches (prefixes were added to some internodes based on their taxonomic position). These labels are unique numbers that are referred to by the internode column of the table. **(B)** The table summarizes the compatibility between the species tree and each of the other trees on internode level (the two internodes with insufficient support in the species tree are marked with asterisks). All insufficiently supported (BS ≥70) internodes were removed from the trees. Internode compatibility can be supported (S), absent (A) or conflicted/discordant (C). Internodes are ordered as they appear on the tree with the exception of ones that correspond to major clades. FFSC, *F. fujikuroi* species complex; The “African” (without *F. acutatum*), American & Asian clade of the FFSC; FOSC, *F. oxysporum* species complex). Column notations: nuc, concatenated nuclear genes; mt-conserved, conserved region of the mitogenome; mt-coding, concatenated coding sequences of the mitogenome (tRNA, rRNA, and protein coding); mt-cds-all, mitochondrial protein coding sequences; mt-cds-core, same as mt-cds-all, except *rps3* and *orf151* are not included; single genes have a prefix nuc for nuclear and mt for mitochondrial.

In order to get a robust estimate of the phylogeny of the analyzed strains, a concatenated alignment was used containing seven nuclear genes (*act, tub2, cal, rpb1, rpb2, tef1a*, and *tef3*) and the conserved region of the mitochondrial genome (excluding the large variable region and introns). The species tree (Figure 1) was estimated with maximum likelihood algorithm and 1,000 ultrafast bootstrap replicates using IQ-tree (Nguyen et al., 2014). Most of the clades on the tree received high bootstrap support except for two terminal branches (shown as Af32 and O27 in Figure 1). Interestingly, *F. acutatum* appeared as an early branching species within FFSC, instead of grouping with the other members from the African clade. In this study, we refer to the clade containing all the African clade member species except for *F. acutatum* as the “African” clade (shown as Af10 in Figure 1), in order to distinguish it from the African clade *sensu* (O'Donnell et al., 1998).

To identify conflicts between the species tree and trees based on subparts (concatenated nuclear genes, the conserved region of the mitogenome, concatenated mitochondrial coding sequences, concatenated protein coding sequence of the mitogenome, single nuclear and mitochondrial genes) of the alignment used for the estimation of the species tree, each tree was compared to the species tree. For the analysis, only internodes/clades that had at least 70% bootstrap support were considered, the rest were collapsed. Figure 1 summarizes the results of this comparison by showing whether a given internode/clade (i) is present (S in Figure 1) with sufficient support (bootstrap ≥70) in the given tree, (ii) is contradicted (C in Figure 1) by a sufficiently supported clade, or (iii) neither (absent or insufficiently supported in the tree; (A in Figure 1). The FFSC, “African,” American, Asian clades are supported by all concatenated trees, and show conflict with no more than one nuclear and a few mitochondrial gene trees. The FOSC clade was supported by three and conflicted by two nuclear single gene phylogenies. Two of the mitochondrial locus phylogenies (*atp8* and *nad4L*) lack well supported clades due to low levels of sequence variation in the alignments. All other phylogenies show at least one conflict (or discordance) with the species tree. These conflicts show the lack of concordance between the different gene genealogies which may be due to ancestral incomplete lineage sorting during the evolution of these groups or to horizontal gene transfer or the combination of the two.

In addition to comparing the species tree with individual phylogenies, the African clade *sensu* (O'Donnell et al., 1998) was also compared. It is contradicted by the tree based on the conserved region of the mitogenome, but supported by the rest of the concatenated trees. From the nuclear gene trees, *tub2* and *rpb1* support the clade, while the other nuclear gene trees are in conflict with it. Interestingly, *tub2* and *rpb1* are the two nuclear markers that contradict the FOSC clade. From the mitochondrial gene trees, *cox1, nad4*, and *nad6* support the clade, while *cox2, cox3, nad1, rns, rnl*, and *rps3* are in conflict with it. The clades of the species tree and the African clade were also tested using the Shimodaira-Hasegawa (SH) and the approximately unbiased (AU) tree topology tests implemented in IQ-tree (Table S1). The SH test has revealed that the only data set that significantly rejects the African clade is the conserved region of the mitogenome. The early branching of *F. acutatum* is significantly rejected by the *tub2* and *rpb1* genes and two of the concatenated mitochondrial data sets in the SH test. The AU test found more conflicts significant than the SH test. The AU test rejected the monophyly of the African clade based on the *rpb2* gene, which was the only nuclear gene that supported the early branching of *F. acutatum*.

Although the phylogenetic analysis based on the concatenated data set resulted in a well resolved tree with highly supported branches, examining the individual loci revealed several conflicts between the phylogenies that may indicate incomplete lineage sorting or horizontal gene transfer events in the evolution of the FFSC. Despite the conflicts in basal branches, all the phylogenies clearly separate FFSC and FOSC.

### 3.2. Mitochondrial Genome Assembly and Annotation

The mitogenomes of all strains were successfully assembled into single contigs with overlapping sequences at ends indicating circular topologies. Overlapping sequences were clipped before annotation and further analysis. The mitogenomes encoded for the expected set of 14 “standard” mitochondrial polypeptide-encoding genes, a set of tRNA coding genes, two rRNA genes, and *rps3* located in an intron of the *rnl*. In addition, all genomes contained orf151 that has no functional prediction, and the large variable (LV) region. In accordance with earlier findings, gene content and order was conserved between species, and all genes and ORFs had the same orientation.

**Figure 2:**
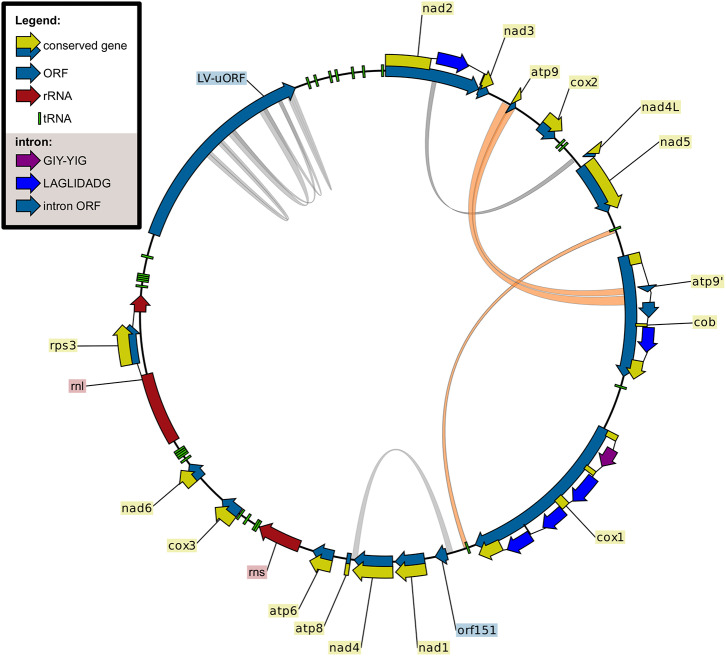
Mitochondrial genome of *Fusarium sacchari* CBS 147.25. Genetic map created using CLC Sequence Viewer. Repeats larger than 100 bp were visualized using Circos, duplication of the *atp9* region into cob-i393 and the two copies of *trnR(tct)* are shown in orange.

**Figure 3:**
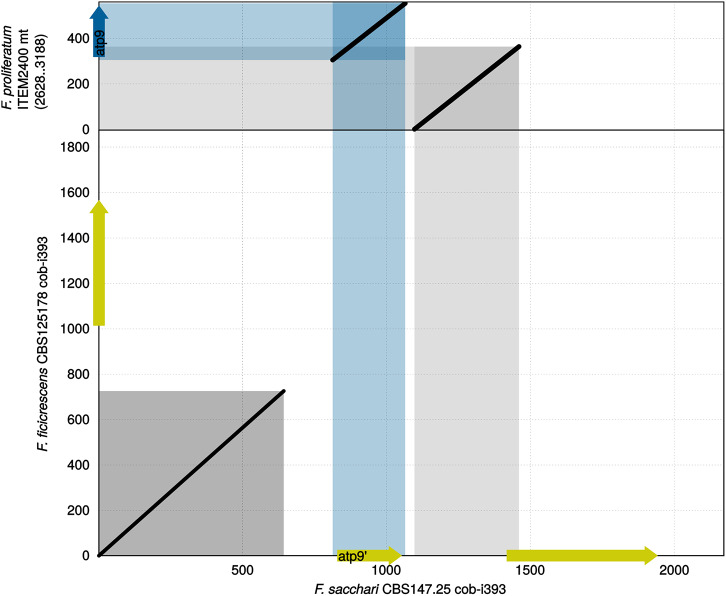
Sequence comparison of cob-i393 of *F. sacchari* against cob-i393 of *F. ficicrescens* and mitogenome segment of *F. proliferatum* containing *atp9*. Conserved *atp9* shown as a blue arrow, while intronic coding sequences are shown as yellow arrows.

In total, 24 intron sites were found in the current data set in the following genes (Table 1): *atp6* (1), *cob* (4), *cox1* (11), *cox2* (1), *cox3* (1), *nad1* (1), *nad2* (2), *nad5* (2), and *rnl* (1). The only intron found in all strains analyzed was the intron of *rnl* harboring the *rps3* gene. The major clades of FFSC show different patterns of intron content: the American clade is intron rich, the Asian clade has a low intron content, while the African clade shows great variation. In the Asian clade, strains contain only the *rnl* intron, with the exception of *F. sacchari*. Interestingly, one of the introns of *cob*, cob-i393, in the mt sequence of *F. sacchari* contains a second copy of *atp9* (Figures 2, 3). The analysis of this duplication is discussed in the next section.

One of the tRNA genes, *trnR(tct)*, was found to be duplicated in all strains analyzed (Figure 2). The duplicated gene shows great conservation, only two sequence variants with a single SNP difference were found in the genomes of the 36 strains. *F. redolens* contained two copies of variant A, while 32 out of 36 strains contained two copies of variant B. Curiously, *F. acutatum, F. commune* and *F. sacchari* contained both variants A and B of *trnR(tct)*. This might be the result of outcrossing of these groups with more distantly related species that contain variant A or it may be due to incomplete lineage sorting.

#### 3.2.1. Duplication of *atp9* Into cob-i393 Intron

Comparing the sequence of cob-i393 intron of *F. sacchari* with that of the other species has revealed conservation of the first 0.7 kbp. The greatest similarity to the *F. sacchari* sequence was shown for the *F. ficicrescens* sequence (Figure 3). The region showing similarity did not contain any putative genes; however, MFannot and RNAweasel identified the intron based on this segment as a group ID intron, which matches the prediction for the other cob-i393 introns. Other ORFs identified in the cob-i393 introns of *F. sacchari* and *F. ficicrescens* had no functional annotation, neither CD-search nor IntreProScan did produce hits. The rest of the cob-i393 sequence of *F. sacchari* showed no similarity to intronic sequences found in the current data set.

The cob-i393 intron of *F. sacchari* contains a duplicate of *atp9* followed by a duplication of the upstream region of *atp9* and the first 24 bp of *atp9* (Figure 3). The *atp9* duplicate contained a codon insertion near the C-terminus and 3 SNPs, which were not found in any of the *atp9* sequences examined in this study. Interestingly, the two duplicated sequence regions (*atp9* and its upstream region) showed greater similarity to other Asian clade strains than to *F. sacchari*. The corresponding regions are identical for all other Asian clade strains.

In conclusion, the duplicate of *atp9* and its upstream region found in the cob-i393 intron of *F. sacchari* has have probably originated from *F. sacchari* or another Asian clade species. During or after the duplication the sequence has mutated.

### 3.3. Large Variable Region

Most of the FFSC species analyzed contained variant 1 of the large variable (LV) region, typical of *Fusarium* spp. However, variant 2, which was previously reported only for *F. oxysporum*, was also found dispersed over the species complex in all three major clades: “African,” American, and Asian. Furthermore, *F. begoniae* contained a LV-region that was a combination of variants 1 and 2.

#### 3.3.1. Hybrid LV-Region in *F. begoniae*

**Figure 4:**
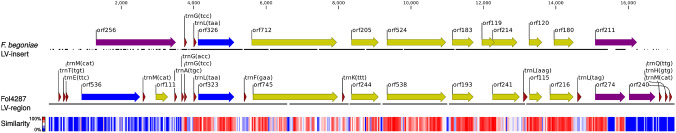
Alignment between the insert in the LV region of *F. begoniae* and variant 2 of *F. oxysporum* Fol4287. Black lines: aligned sequences, gaps in the alignment are shown as gaps in the black lines; red triangles: tRNA genes; yellow arrows: ORFs; blue arrows: LAGLIDADG endonucleases; purple arrows: GIY-YIG endonucleases. Sequence similarity is indicated in color scale based on 30 bp segments. The scale above indicates position within the alignment.

The first 895 bp and the last 11,807 bp of the 25,064 bp long LV-region appeared to be an ortholog of variant 1 (Figure S1), while the 12,462 bp long sequence between these two segments showed high sequence similarity to a major part of variant 2 (Figure 4 and Figure S2). These results indicate that the sequence segment similar to variant 2 represents a putative insert into the canonical variant 1 sequence.

The putative insert is located between two tRNA genes: the second copy of *trnM(cat)* and *trnG(tcc)*. Interestingly, the nucleotide preceding the insertion site is a thymidine and the last nucleotide of the insert is a guanosine which is the same as for typical mitochondrial introns. Although rnaweasel did not identify any intron specific motifs in the insert sequence both terminal regions of the insert contain ORFs that were predicted to be GIY-YIG homing endonucleases. The putative GIY-YIG endonuclease located near the upstream terminus of the insert showed no homology to any other sequence included in the current study, although it produced a partial hit with BLASTN to an ORF (orf275) in the mitogenome of *Beauveria caledonica* (GenBank accession: KT201150). The ORF is located in the intergenic region between *cox3* and *nad6* in *B. caledonica*. The putative GIY-YIG endonuclease located near the downstream terminus of the insert showed partial similarity to one of the GIY-YIG endonuclease found in the downstream region of variant 2 (Figure 4). The insert contains mostly ORFs with unknown functions, no hits were obtained with CD-Search or InterProScan, the size and positions of these ORFs are similar to the ones found in variant 2 sequences. Both the insert and variant 2 have the same two tRNA genes located upstream of a putative LAGLIDADG endonuclease. However, the insert contains no other tRNA genes in contrast to variant 2 of the LV region.

The inserted region shows several characteristics that are similar to mitochondrial regions: the preceding thymidine and the terminal guanosine nucleotide, also the presence of homing endonuclease genes. In addition, one of the endonucleases is similar to homing endonuclease that is located in intergenic region of *B. caledonica*. The insertion may have occurred following the same mechanism as introns are spread with a homing endonuclease that may recognize an intergenic target.

#### 3.3.2. LV Variant 2 Shows Signs of Horizontal Transfer

To investigate whether the distribution of variants 1 and 2 within the FFSC is the result of incomplete lineage sorting or horizontal transfer of sequences, we compared the pairwise sequence distances based on the LV variant sequences and those based on the conserved part of the mitochondrial genomes, which excluded the LV-region and intron sequences. The two distances, based on variant sequence and based on the conserved region, were plotted against each other using a scatter plot. A trend line, representing the correlation between the two distances, was estimated for each of the plots. For this analysis three alignments were created: for variant 1, for variant 2, and for the conserved region. The putative insert in the LV-region of *F. begoniae* was excluded from this analysis, by removing it from the LV sequence; the resulting sequence was included as a variant 1 sequence.

**Figure 5:**
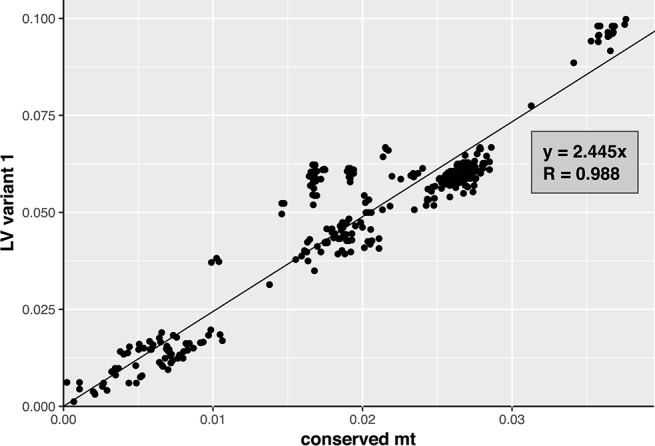
Variant 1 of the LV region plotted against the mean genetic distance. The plot of genetic distance between strains based on variant 1 of LV-region against conserved mt region with trend line. Function of the trend line and *R* are shown on the plot.

**Figure 6:**
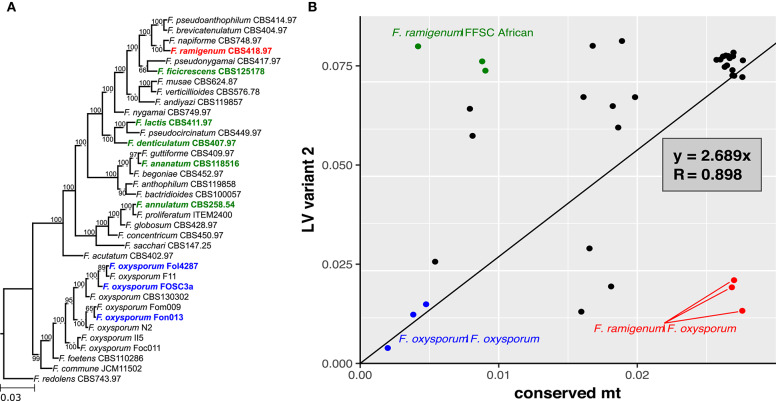
LV region of *F. ramigenum* is most similar to that of *F. oxysporum*. **(A)** Species tree with bootstrap support, strains with variant 2 of the LV region are shown in boldface. **(B)** The plot of genetic distance between strains based on variant 2 of LV-region against conserved mt region with trend line. Function of the trend line and *R* are shown on the plot. FFSC vs. *F. ramigenum* are shown in green, while FOSC vs. *F. ramigenum* is shown in red. The three FOSC comparisons are shown in blue.

The pairwise genetic distances estimated based on variant 1 sequences showed good correlation with those based on the conserved mt region (*R* = 0.988; Figure 5). In contrast, correlation between the pairwise genetic distances estimated based on variant 2 and the conserved region showed weaker correlation (*R* = 0.898; Figure 6B). The variant 2 sequence of *F. ramigenum* was more similar to that of strains belonging to the FOSC than to strains belonging to the FFSC (Figure 6B). The topology of the maximum likelihood tree calculated based on variant 2 (Figure S3) showed several conflicts with the conserved region or the species tree, most notably: (i) *F. ramigenum* was clustered inside the FOSC with high support, and (ii) *F. annulatum* (Asian clade) and *F. ficicrescens* (“African” clade) grouped together in a well-supported (bootstrap=100) clade separate from the rest of the FFSC strains.

In conclusion, the variant 1 of the LV region appears to follow the evolution of the conserved parts of the mitogenome, while the variant 2 shows several deviations from the expected pattern. Variant 2 is distributed across the FFSC and FOSC and its genealogy deviates from those of other markers that suggests the pattern is the result of horizontal transfer, most probably due to introgression.

## 4. Discussion

Three biogeographic clades (African, American, and Asian) have been described within the *F. fujikuroi* species complex (FFSC) based on phylogenetic data (O'Donnell et al., 1998). Although multilocus sequence analysis supports the recognition of the three clades, several genetic markers produce topologies that appear to contradict this grouping, such markers include the ITS2 region (O'Donnell et al., 1998, 2000) and the fumonisin biosynthetic gene (FUM) cluster (Proctor et al., 2013). Comparative mitochondrial genome analyses have indicated possible recombination events in the evolutionary history of the *F. fujikuroi* species complex (FFSC) based on conflicting single gene tree topologies (Fourie et al., 2013, 2018). In this study, a large set (24 species) of FFSC and a representative set of FOSC strains were sequenced to compare their mitochondrial genome, and to contrast phylogenetic results of concatenated regions and individual genes, both nuclear and mitochondrial, in order to investigate the evolutionary relationship between and within these groups.

The phylogenetic analysis indicated that *F. acutatum* clusters separately from the three biogeographic clades, while it should group within the African clade (O'Donnell et al., 1998). The monophyly of the African clade was supported by only five of the markers, but was conflicted by eleven loci analyzed. The weak support for the clustering of *F. acutatum* within the African clade is in agreement with earlier findings (O'Donnell et al., 2000). However, the other two clades (American and Asian) were robustly supported by the majority of loci, and even the “African” clade (African clade = *F. acutatum* ∪ “African” clade) was robustly supported. The “African” clade contained the largest number of clades with extensive conflicts with the individual gene trees. In addition, the “African” clade had the most diverse intron pattern, while the other two clades had a more homogeneous intron distribution. Both of these facts suggest that incomplete lineage sorting or recombination is confounding the phylogenetic reconstruction in this group.

The comparative analysis of the mitochondrial genome sequences provided further evidence for deviation from the classical tree-like model of evolution for the FFSC. Variant 2 of the LV-region was found in representatives of all three major clades of the FFSC. This variant was previously known only for clades 1 and 2 of the *F. oxysporum* species complex (FOSC), where the most probable explanation for the distribution of the variant across both clades was shown to be incomplete lineage sorting (Brankovics et al., 2017). In order to identify whether the distribution of the variant within the FFSC can be explained by horizontal transfer or incomplete lineage sorting, the genetic distance calculated based on the LV variants was compared to that calculated based on the conserved region of the mitogenome. The genetic distances based on variant 1, the predominant variant in the genus, showed strong correlation with those based on the conserved regions. However, the results for variant 2 significantly deviated from the pattern expected for vertical inheritance. The variant 2 sequence of *F. ramigenum* showed greater similarity to variant 2 sequences of FOSC strains than to that of other FFSC strains, indicating horizontal transfer of this region between members of the two species complexes. Besides horizontal transfer between the two species complexes, the sequence evolution of variant 2 is suggestive of horizontal transfer between the different clades within the FFSC.

Interfertility of species belonging to the Asian clade of the FFSC has been demonstrated under laboratory conditions (Leslie et al., 2004). The viability of the progeny of interspecies crosses was shown to be significantly lower than for within species crosses, indicating only partial genetic incompatibility between these species. Although the genetic distance between *F. oxysporum* and FFSC is greater than the genetic distance between any two FFSC species, both *F. oxysporum* and FFSC species have 12 core chromosomes, which show large levels of synteny to each other (Ma et al., 2010). This similarity on chromosome level may allow low levels of cross-fertility even between the two species complexes. Although *F. oxysporum* is usually considered asexual, based on the phenomenon of horizontal chromosome transfer between strains and comparative mitogenome analysis it supposed to have a cryptic sexual or (para)sexual cycle (Brankovics et al., 2017). The possibility of (rare) out-crossing between the two complexes is supported by phylogenetic data of the ITS2 region (O'Donnell et al., 1998) and of the FUM cluster (Proctor et al., 2013). In the case of ITS2, both species complexes have been shown to have two divergent types of the region (O'Donnell et al., 1998). While for the FUM cluster, the two FOSC strains that carry the cluster group within the FFSC based on FUM gene trees (Proctor et al., 2013). These findings show that introgression between the FFSC and FOSC has played an important role in the evolutionary history of these two complexes.

In the mitochondrial genomes, the conserved genes show complete synteny and the coding sequences show very high levels of sequence similarity, whereas the nuclear chromosomes show occasional rearrangements. Thus, recombination between mitogenomes would be less likely to lead to lower fitness than nuclear recombination, because it is less likely to lead to rearrangements or deletions. Cytoplasmic fusion without karyogamy between hyphae of FFSC and FOSC strains could allow for mitochondrial recombination as well as transfer of genetic information between the nuclei through mobile elements or other genetic mechanisms. Accordingly, mitogenomes could be used as indicators for detecting interspecies gene flow.

Besides the phylogenetic value of the mitogenomes assembled for this study, their comparative analysis provides insights into the evolution of these organellar genomes. Analysis of mitogenomes of the FOSC has already indicated that homing endonucleases (HE) may have played an important role in the origin of variants 2 and 3 of the LV region (Brankovics et al., 2017). Homing endonucleases are frequently associated with group I mitochondrial introns; they are able to specifically cleave double stranded DNA and introduce their genes into the target region (Burt and Koufopanou, 2004). In the current study, a putative insert was identified in the LV region of *F. begoniae* that contained HE genes at both termini, providing further support for the importance of HE genes in mitochondrial genome rearrangements. Since HEs have a driving role in gene conversion events, the most likely sites for introgression events are the target sequences of HEs and their vicinity.

Another interesting finding of the mitogenome analysis is the identification of the duplication of *atp9* into an intron of *cob* in *F. sacchari*. The resulting intron contains no HE genes, which is similar to the intron in *rnl* that contains the *rps3* gene only. The translocation of *rps3* into the intron of *rnl* happened in the ancestor of the *Pezizomycotina* (Ghikas et al., 2006). The duplication of *atp9* indicates that the same or similar mechanisms are still active on the mitogenome of the FFSC. The intronization of genes and intergenic segments, like that of *atp9* and its upstream intergenic region, may allow the evolution of rearranged regions that afterwards could replace the original region through recombination. This could be the mechanism behind the evolution of the new variants of the LV region. The intronic origin would also explain why HE genes are present in this region, since they are typically intron encoded.

## 5. Conclusions

Complete mitogenome sequences can provide a robust basis for phylogeny. In contrast, the duplication of *atp9* suggests that single mitochondrial genes may lead to inaccurate phylogenetic estimates, due to possible paralogs.

Mitogenomes could be used as indicators for detecting interspecies gene flow, since mitogenomes show greater levels of conservation and synteny than nuclear regions. Introgression among and between members of *Fusarium oxysporum* and *F. fujikuroi* species complexes has played an important role in the evolutionary history of these two complexes, which has to be taken into consideration when analyzing gene genealogies for these groups.

## Data Availability Statement

The datasets generated for this study can be found in the NCBI's GenBank database (MT010857-MT011064) and in the SRA (NCBI/ENA) database (PRJEB38038).

## Author Contributions

BB, AD, TL, and CW designed the study. AD, TL, and CW sequenced large part of the strains used for this study. BB carried out the analysis. BB wrote the first draft. All authors helped shaping the manuscript and approved the final version of the manuscript.

### Conflict of Interest

The authors declare that the research was conducted in the absence of any commercial or financial relationships that could be construed as a potential conflict of interest.

## Supplementary Material

Figure S1 LV region of *F. begoniae* and of *F. anthophilum* (variant 1). Gray area represents the insert region in the LV-region of *F. begoniae*.

Figure S2 LV region of *F. begoniae* and of *F. oxysporum* Fol4287 (variant 2). Gray area represents the insert region in the LV-region of *F. begoniae*.

Figure S3 Unrooted maximum likelihood tree based on LV variant 2 sequences. Bootsptrap values (based on 1,000 replicates) are indicated below the branches.

Table S1 The *p*-values for the topology constrain analysis. The internodes are the same that were used for Figure 1B with the addition of the African clade.
