# Supplementary material for: Detecting Introgression Between Members of the Fusarium fujikuroi and F. oxysporum Species Complexes by Comparative Mitogenomics
Source: Front Microbiol. 2020 Jun 3;11:1092. doi: 10.3389/fmicb.2020.01092 (PMC7285627; doi:10.3389/fmicb.2020.01092)
Supplement: Figure S1 — LV region of F. begoniae and of F. anthophilum (variant 1). Gray area represents the insert region in the LV-region of F. begoniae. [file Data_Sheet_1.PDF]

Supplementary Material

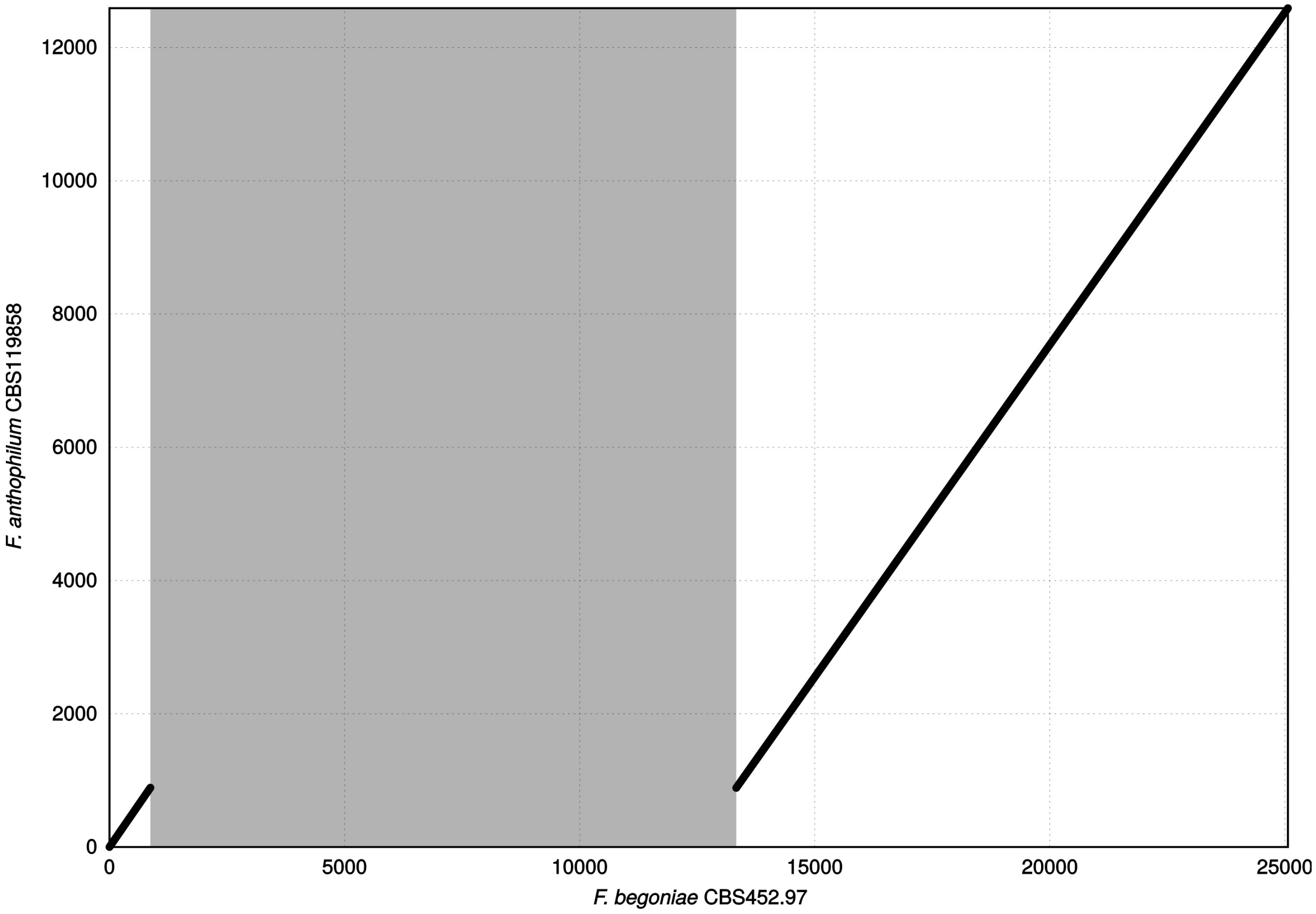

Figure S1. LV region of *F. begoniae* and of *F. anthophilum* (variant 1). Grey area represents the insert region in the LV-region of *F. begoniae*

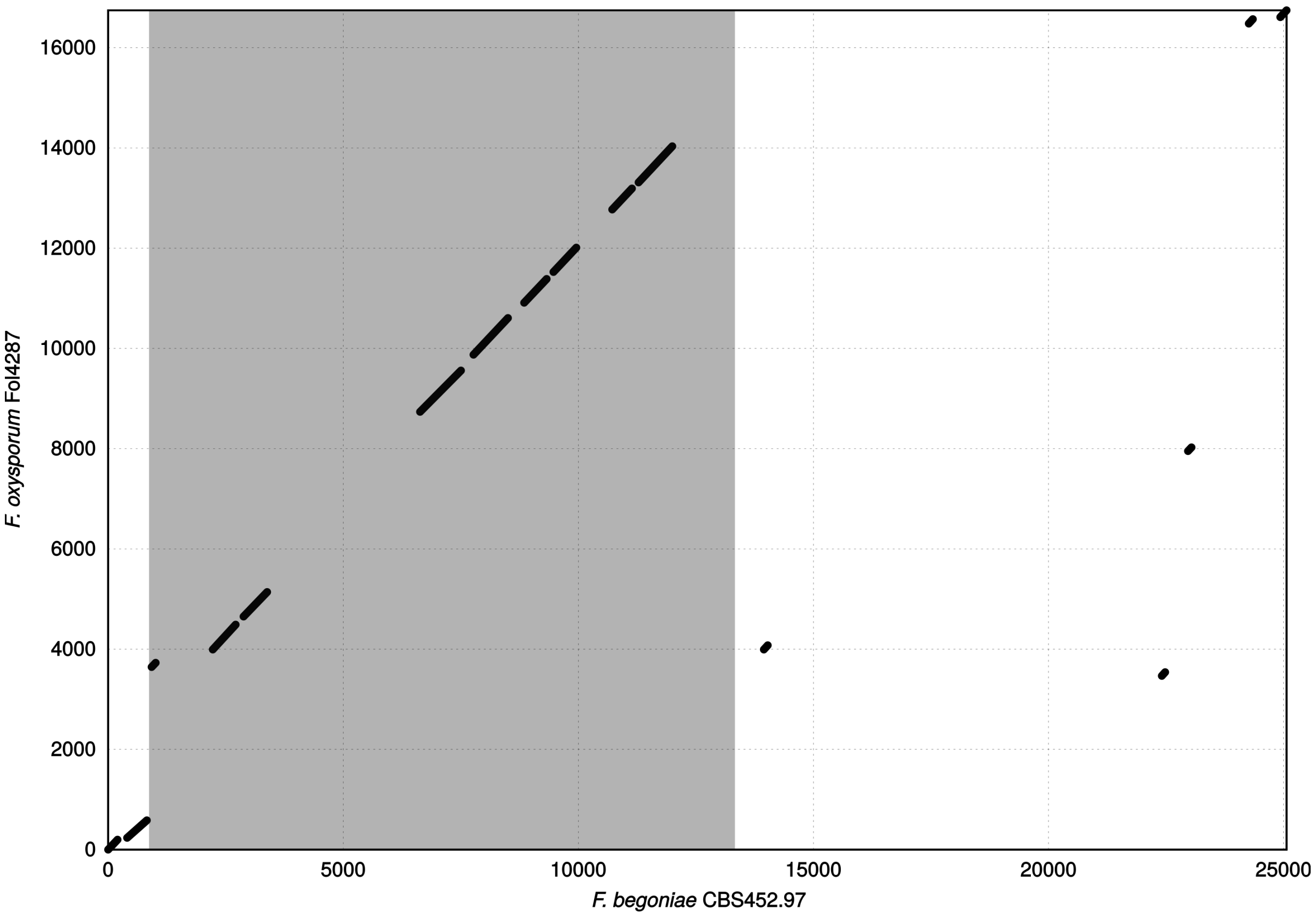

Figure S2. LV region of *F. begoniae* and of *F. oxysporum* Fol4287 (variant 2). Grey area represents the insert region in the LV-region of *F. begoniae*

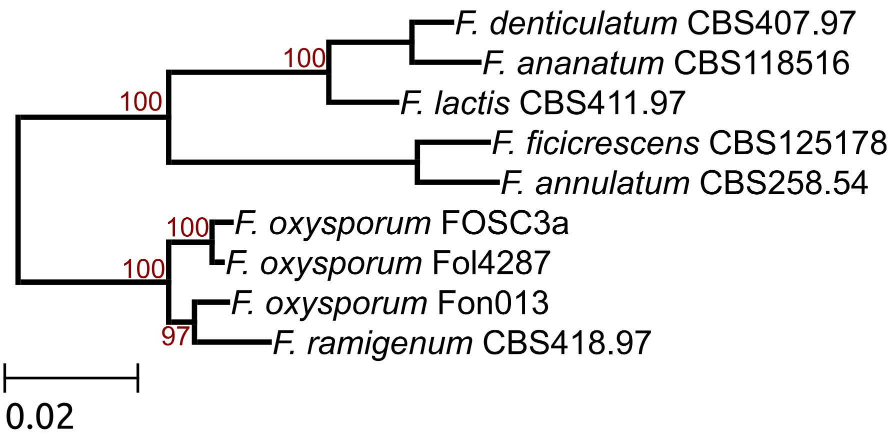

Figure S3. Unrooted maximum likelihood tree based on LV variant 2 sequences. Bootsptrap values (based on 1000 replicates) are indicated below the branches.
